# On the value of seasonal mammals for identifying mechanisms underlying the control of food intake and body weight

**DOI:** 10.1016/j.yhbeh.2014.03.009

**Published:** 2014-06

**Authors:** Francis J.P. Ebling

**Affiliations:** School of Life Sciences, University of Nottingham Medical School, Queen's Medical Centre, Nottingham NG7 2UH, UK

**Keywords:** Appetite, Food intake, Body weight, Thyroid hormone, Season, Photoperiod

## Abstract

This article is part of a Special Issue “Energy Balance”.

Seasonal cycles of adiposity and body weight reflecting changes in both food intake and energy expenditure are the norm in mammals that have evolved in temperate and polar habitats. Innate circannual rhythmicity and direct responses to the annual change in photoperiod combine to ensure that behavior and energy metabolism are regulated in *anticipation* of altered energetic demands such as the energetically costly processes of hibernation, migration, and lactation. In the last decade, major progress has been made into identifying the central mechanisms that underlie these profound long-term changes in behavior and physiology. Surprisingly they are distinct from the peptidergic and aminergic systems in the hypothalamus that have been identified in studies of the laboratory mouse and rat and implicated in timing meal intervals and in short-term responses to caloric restriction. Comparative studies across rodents, ungulates and birds reveal that tanycytes embedded in the ependymal layer of the third ventricle play a critical role in seasonal changes because they regulate the local availability of thyroid hormone. Understanding how this altered hormonal environment might regulate neurogenesis and plasticity in the hypothalamus should provide new insight into development of strategies to manage appetite and body weight.

## Highlights

• Seasonal cycles reveal novel mechanisms underlying control of food intake.
• Hypothalamic tanycytes control seasonal changes in ingestive behavior.
• Tanycytes regulate thyroid hormone transport and availability in the hypothalamus.
• Thyroid hormone may regulate mechanisms important in initial development.
• The adult seasonal hypothalamus is a region of structural and functional plasticity.

## Introduction

**Fig. 1:**
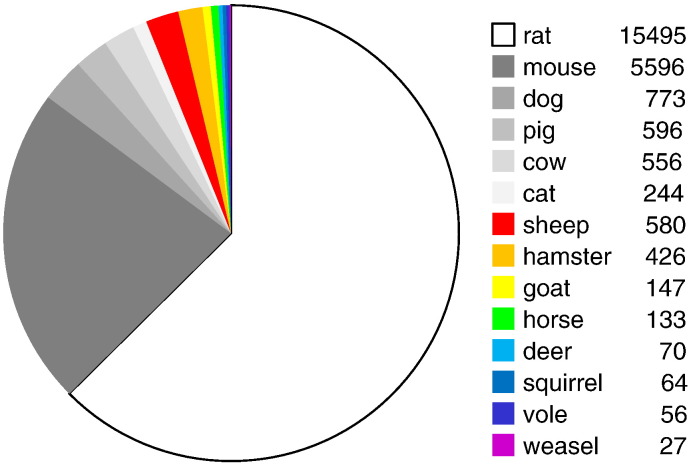
Percentage of citations in PubMed (August 2013) using the search terms “appetite” OR “food intake” AND the animal group indicated. Absolute numbers of citations are indicated to the right of the animal group. Note that citations with “rat” or “mouse” as a search term comprise nearly 80% of the total, and seasonal mammals (in color) only constitute ~ 6% of the total. 28,895 citations using “human” or “man” with these search terms were also identified.

Given the high international prevalence of obesity and the substantive costs of management of the associated co-morbidities, the need to develop pharmacological strategies to reduce appetite and support weight-control regimes has never been greater. As of 2013, there are no centrally-acting drugs that can be prescribed in the USA or Western Europe for this purpose, the most recently available compounds sibutramine (a monoamine reuptake inhibitor) and rimonabant (an endocannabinoid receptor antagonist) having been withdrawn because of adverse side effects (James et al., 2010; Rothman and Baumann, 2009). Clearly there is a huge need to understand better the complex behavioral mechanisms that underlie our motivation to eat in order to identify feasible drug targets, but our use of animals to achieve this is hugely skewed toward a few ‘model’ species. A brief analysis of the PubMed database in August 2013 using the search terms “appetite” OR “food intake” AND more specific mammalian species or genera revealed that only approximately 6% of all manuscripts were based on studies in seasonal species, of which sheep and hamsters were the most common (Fig. 1).

Of the remaining 94% of studies, approximately two-thirds were studies in rats, and about a quarter were studies in mice (Fig. 1). Even acknowledging the biases inherent in searching for studies utilizing seasonal species in a biomedical database, this simple analysis suggests that our understanding of the biology of appetite control is largely restricted to a few ‘model’ species. The limitations of using laboratory mice and rats as experimental models to understand human mechanisms or to predict drug efficacy has been reviewed in depth elsewhere; under standard conditions most strains are “sedentary, overweight, insulin resistant, hypertensive and prone to premature death” (Martin et al., 2010). There is no question that analysis of single gene mutations in mice and rats (e.g. *ob*/*ob*) that have arisen spontaneously or arising from genetic manipulation (e.g. the plethora of mutations of the melanocortin system) identify fundamental mechanisms involved in control of energy balance, but it is equally apparent that relatively few cases of clinical obesity reflect single gene mutations (Farooqi and O'Rahilly, 2006). The focus of this review is therefore to highlight how studies in mammals that undergo natural seasonal cycles of altered appetite and of fat deposition and catabolism have led to the identification of novel pathways that were missed in studies of intensively-bred conventional laboratory species. However, it is also interesting to consider the evidence that *Homo sapiens* is a seasonal species, particularly with regard to annual patterns of reproduction (Roenneberg and Aschoff, 1990a,b), mood and affect (Partonen and Lönnqvist, 1998), and immune function and susceptibility to illness (Nelson, 2004). While it is difficult to disentangle the relative contributions of social and cultural influences from the underlying true seasonal rhythmicity (Bronson, 2004; Mersch et al., 1999), increasing our understanding of the basic biological processes underlying seasonal rhythms is likely to have beneficial impact of human wellbeing where modern life styles conflict with our seasonal nature (Foster and Kreitzman, 2010).

### Seasonal strategies

Seasonal cyclicity of reproductive and metabolic physiology coupled to complementary behaviors is almost ubiquitous among organisms that evolved in non-equatorial latitudes. Reproduction is an energetically costly process, and mammals have evolved strategies to give birth and raise young at times of year that favor survival and nutritional support of lactation and growth of the offspring. For terrestrial mammals this is usually spring, but as gestation lengths vary hugely between species, and because food availability and storage mechanisms also vary, a wide variety of different seasonal strategies are found. In mammals with short gestation periods of a few weeks such as hamsters, deer mice, shrews and voles, the neuroendocrine activation of the reproductive axis occurs in late winter/early spring at the time when day lengths are increasing. In larger species with longer gestation periods, for example sheep (5 months), goats and red deer, neuroendocrine activation occurs during the fall when day lengths are decreasing. Superficially, species can be divided into “long-day” and “short-day” breeders, but this nomenclature can be misleading, for example some species such as pine martens and stoats have short gestation periods but nevertheless breed in late summer when day length is decreasing because they then have a prolonged period of delayed implantation of the blastocyst, leading to births in the following spring (Amstislavsky and Ternovskaya, 2000). Despite different reproductive cycles between “long-day” and “short-day” breeders, there may be commonality of timing of other cycles. Almost all mammals studied to date show a summer rise in prolactin secretion which co-ordinates changes in growth and molting of fur and wool (Lincoln, 1990). Most species will also gain body weight in the summer as they store energy as fat depots (Bartness et al., 2002), and many use strategies such as torpor and hibernation to conserve energy in winter (Wilcox and Willis, 2014), this may be combined with programmed winter hypophagia, thus only limited energy is spent on foraging for diminished food reserves (Heldmaier et al., 1989). However the precise timing and inter-relationship of seasonal cycles of appetite, fattening and reproductive activity is species specific, perhaps reflecting the trade-offs that have evolved between appetitive behaviors underlying acquisition of calories versus those promoting reproduction (Schneider et al., 2013). Moreover, even within species there may be polymorphisms in the inter-relationships of seasonal cycles of metabolic rate, feeding, activity and fertility [see White et al., 2014 for review].

### Leptin and seasonal body weight cycles

A classic series of lesion studies in the mid part of the 20th century identified the hypothalamus as a key homeostatic structure regulating feeding, satiety and energy balance (Anand and Brobeck, 1951; Hetherington and Ranson, 1940) as reviewed by Elmquist et al. (1999). Along with the brainstem, it detects circulating signals such as leptin and insulin relating to the condition of energy stores within the body, levels of energy metabolites such as glucose and fatty acids, and signals such as Ghrelin and cholecystokinin relating to the activity of the gastrointestinal tract, then integrates this information to affect both behavior and physiology. The cloning of leptin (Zhang et al., 1994) and the subsequent identification of leptin receptors initiated a huge leap forward in identifying specific hypothalamic pathways subserving these functions in mice and rats. Accordingly, under negative energy balance conditions, peripheral leptin concentrations are reduced, which promotes increased orexigenic gene expression in the mediobasal (e.g. NPY, AgRP) and lateral hypothalamus (e.g. orexin, MCH) and decreased anorectic gene expression (e.g. POMC, CART, TRH) (Friedman, 2009). Thus, in the short-term, decreased leptin production in response to reduced caloric availability or starvation engages food-seeking behaviors, and correspondingly promotes energy-saving adaptations such as decreased metabolic rate and torpor. Conversely, treatment of rodents in a leptin-deficient state with synthetic leptin suppresses food intake (Friedman, 2009). However, in seasonal mammals circulating leptin levels naturally decrease as intra-abdominal fat depots decrease in the short days preceding winter. Unlike the situation in rats and mice, there is a “leptin paradox (Morgan et al., 2006)”; that is, the seasonal decrease in leptin production is *not* perceived as a ‘starvation’ signal: it does not trigger a compensatory response, rather it is associated with decreased appetite. Moreover, the collective finding of many research groups is that those genes identified above as being leptin-responsive do not show clear seasonal or photoperiodically-driven changes in expression despite clear changes in circulating leptin levels (Mercer et al., 1997; Reddy et al., 1999; Robson et al., 2002; Rousseau et al., 2002). At a mechanistic level, studies in the Siberian hamster reveal that they become more sensitive to leptin feedback in the short day lean state, and relatively leptin-resistant in the long day fat state (Atcha et al., 2000; Rousseau et al., 2002). At the cellular level this leptin-resistance reflects both an increase in SOCS3 [suppressor of cytokine signaling] expression, and a reduction in leptin-induced phosphorylation of STAT3 [signal transducer and activator of transcription 3] (Tups, 2009). These results suggest one of two hypotheses: either long photoperiods directly increases leptin resistance, or they increase energy intake and body fat content which indirectly causes leptin resistance. There is ample evidence for the latter, since during prolonged long-day exposure and in non-seasonal animals, leptin resistance is positively correlated with body fat content (Friedman and Halaas, 1998).

There are two other lines of evidence that indicate that the known hypothalamic peptidergic systems are not at the heart of seasonal control of appetite and body weight. First, treatment of neonatal hamsters with monosodium glutamate causes major lesions of the arcuate nucleus, the principal hypothalamic locus of NPY/AgRP and POMC neurons. Such treatment renders hamsters hypogonadal, and prone to obesity in later life, but importantly it does not disrupt photoperiodic control of growth and body weight (Ebling et al., 1998). Approximately 90% of neurons are destroyed in the arcuate nucleus by this treatment, including corresponding loss of NPY and tyrosine hydroxylase (dopaminergic) neurons, suggesting that such neurons are not critical for seasonal metabolic cycles (Ebling et al., 1998). Correspondingly, massive overexpression of AgRP in the hypothalamus induced by local infusion of a recombinant adeno-associated viral construct containing a AgRP coding sequence under control of a CMV promoter increased appetite and body fat as expected, but did not disturb the normal catabolic response to short-day photoperiods (Jethwa et al., 2010). Collectively, these different experimental strategies lead to the conclusion that the leptin–hypothalamic peptide systems that are so important in body weight homeostasis in traditional laboratory species are not the drivers of seasonal body weight cycles, hence the recent efforts to identify novel mechanisms.

### Seasonal changes in hypothalamic gene expression

A number of studies have used transcriptomic approaches to identify differential gene expression in the hypothalamo-pituitary axis of mammals in seasonally distinct states, for example in Siberian hamsters (Prendergast et al., 2002; Ross et al., 2004), sheep (Dupre et al., 2008) and in a photoperiodic strain of rat (Ross et al., 2011). Two themes have emerged. First, changes in gene expression have generally *not* been observed in the hypothalamic nuclei implicated in short-term and homeostatic control of appetite, namely the arcuate, ventromedial, dorsomedial and paraventricular nuclei. Rather, the main changes in gene expression have been in the dorsomedial posterior arcuate nucleus and in the ventral ependymal cell layer lining the third ventricle. The implication of the latter finding is that seasonal changes in gene expression are occurring in glial rather than neuronal cells. Almost certainly it is the tanycyte population within the ependymal layer that are the key cells involved (Rodriguez et al., 2005). Second, the most consistent cluster of ‘seasonal’ genes are those encoding proteins involved in the transport, metabolism and receptor-mediated signaling of thyroid hormone and retinoic acid. As these signaling pathways are both important in initial brain development, many groups have hypothesized that seasonal cycles in neuroendocrine function reflect plastic changes in the brain, that is, they depend upon re-use of developmental mechanisms.

The most notable seasonal changes in gene expression in the ependyma are those for deiodinase 2 (DIO2) and deiodinase 3 (DIO3). These genes encode enzymes responsible for local tissue metabolism of thyroid hormone. DIO2 promotes conversion of thyroxine (T4) into the biologically active metabolite tri-iodothyronine (T3) and so increases local tissue concentrations of thyroid hormone, whereas DIO3 converts T4 to inactive reverse T3, and also deiodinates T3, so has the contrasting effect of reducing local tissue concentrations of thyroid hormone. Across all mammalian species studied to date, there is a consistent pattern of high levels of DIO2 expression and very low levels of DIO3 expression in long days (Table 1), whereas exposure of animals to short photoperiods tends to reduce DIO2 expression, but markedly upregulates DIO3 expression (Table 1). This pattern has also been observed in photoperiodic strains of lab rats and mice (Table 1), and in comparable structures in other vertebrate groups including birds (Watanabe et al., 2007; Yoshimura et al., 2003) and teleost fish (Nakane et al., 2013), suggesting an ancient phylogenetic origin (Hanon et al., 2008). Photoperiodic regulation of the thyroid hormone transporters MCT8 and organic anion transporter family member 1C1 (Oatp1c1) have also been detected, though short days upregulate MCT-8 but downregulate Oatp1c1, so it is not clear whether these are primary responses to photoperiod or compensatory responses to altered local thyroid hormone concentrations.

Complementing these seasonal changes in elements of the thyroid hormone signaling pathway, in hamsters and a photoperiodic strain of rat, changes in genes encoding elements of the retinoic acid pathway have been detected (Ross et al., 2004; Shearer et al., 2010). This is of considerable interest, given the crosstalk between thyroid hormone receptors (TH) and retinoic acid receptors (RAR) with retinoid x receptors (RXR) in the nucleus (Liu and Brent, 2010). Examples of photoperiodically regulated genes include the retinoic acid transporters, synthetic enzymes (RALDH, retinaldehyde dehydrogenase), binding proteins (cellular retinoic acid binding protein 1 CRBP1 and *CRABP2*), and receptors (Stra6, stimulated by retinoic acid gene 6, RAR, RXRγ). Moreover, a plethora of other changes in gene expression in the ependymal cell layer that might also impact upon hypothalamic structure have been identified, including decreased expression of nestin and the melatonin-related receptor GPR50 in Siberian hamsters in short days (Barrett et al., 2006).

### Photoneuroendocrine pathways

**Fig. 2:**
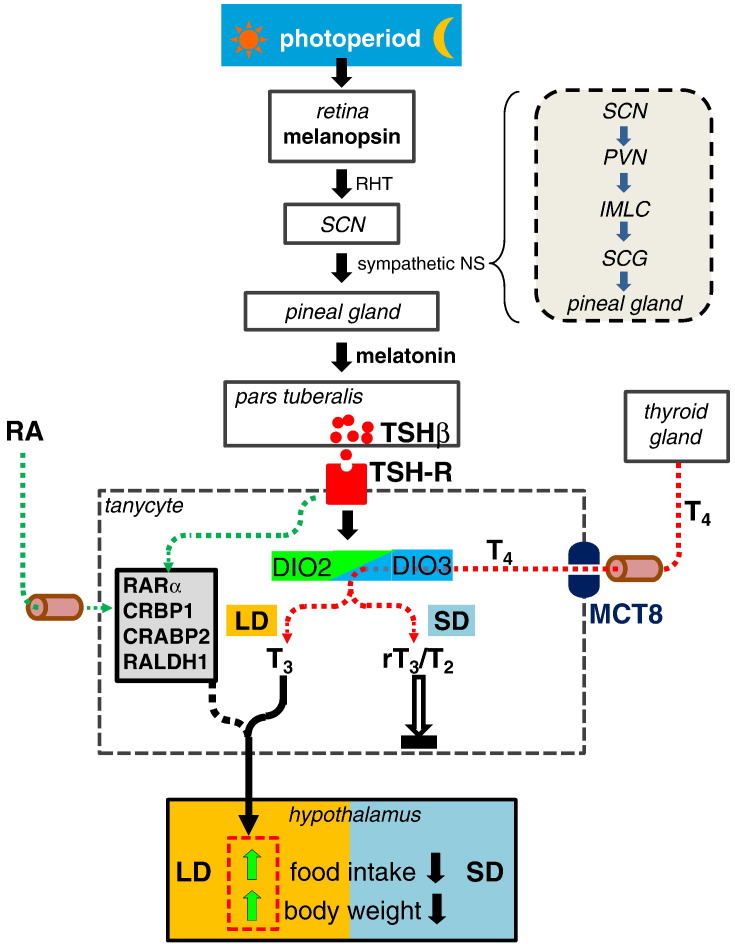
Schematic summary of the photoneuroendocrine control of food intake and body weight. Photoperiodic information detected by melanopsin-expressing ganglion cells in the retina is relayed via the retinohypothalamic tract (RHT) and suprachiasmatic nucleus (SCN) to the pineal gland, thereby regulating the nocturnal duration of melatonin secretion. The box indicates key points of this pathway, SCN efferents project to the paraventricular nucleus (PVN), where presympathetic fibers project to sympathetic cell soma in the intermediolateral column (IMLC) in the spinal cord. Preganglionic fibers terminate in the superior cervical ganglion (SCG), innervating postganglionic fibers which terminate in the pineal gland. Melatonin signals to the *pars tuberalis*, promoting TSHβ production in long photoperiods (LD) that binds to its cognate receptor in tanycytes, and upregulates type 2 deiodinase (DIO2) expression and possibly signaling in the retinoic acid (RA) pathway [RARα: CRBP1: CRABP2: RALDH1:]. Thyroxine (T4) is taken up from the circulation via MCT8 transporters, and in LD is converted by DIO2 to tri-iodothyronine (T3), which exerts local anabolic actions within the mediobasal hypothalamus. In short photoperiods (SD), the long nocturnal duration of melatonin reduces TSHβ production, expression of type 3 deiodinase (DIO3) is upregulated, thus inactive metabolites of T4 such as reverse T3 (rT3) are produced resulting in a catabolic state.

The mechanism by which photoperiod regulates these changes in gene expression in the ependymal cell layer clearly depends upon photoperiodic modification of the pattern of melatonin production in mammals (Fig. 2). Melanopsin-containing retinal ganglion cells detect changes in luminescence, and signal directly to the suprachiasmatic nucleus such that sympathetic control of melatonin production of the pineal gland is increased at night (Fig. 2). As photoperiod changes annually, so does the nocturnal duration of melatonin production, thereby providing a neurochemical index of night length. The likely site of action of melatonin is the *pars tuberalis*, a part of the pituitary stalk that apposes the median eminence of the hypothalamus, and contains a strikingly high density of melatonin receptors in all mammalian species studied to date (Bittman, 1993; Bittman and Weaver, 1990; Morgan et al., 1994; Williams and Morgan, 1988). Indeed, studies using the radioligand 2-iodomelatonin in seasonal mustelids including ferrets (Weaver and Reppert, 1990), mink (Bonnefond et al., 1993) and skunks (Duncan and Mead, 1992) reveal that the *only* location of melatonin binding sites is in the *pars tuberalis*. The complete absence of evidence of 2-iodomelatonin binding in the brain of mustelids supports the view that this tissue is the key site for regulation of seasonal rhythms by melatonin.

The anatomical evidence for the *pars tuberalis* being a critical site of melatonin action is supported by elegant functional studies in a semi-domesticated breed of sheep, the Soay ram. A series of studies demonstrated that surgical transection of the pituitary stalk blocks photoperiodic control of seasonal cycles of reproduction (Lincoln and Clarke, 1998), appetite and body weight (Lincoln et al., 2002), yet leaves photoperiodic control of prolactin secretion and its downstream targets (growth and molting of wool, and horn growth) intact (Lincoln and Clarke, 1994, 1995; Lincoln et al., 2003). Subsequent melatonin treatments were able to resynchronize prolactin cycles but not reproductive or metabolic cycles. The implication is that the reproductive and metabolic axes require an intact *pars tuberalis*–hypothalamus pathway for melatonin to exert seasonal entrainment, whereas the prolactin axis functions independently, probably through direct actions of the *pars tuberalis* on the function of lactotrophs in the *pars distalis* (Dupre et al., 2010).

Consistent with the focus on the *pars tuberalis* as the principal site of melatonin action in regulation of seasonal rhythms, a number of genes known to be involved in circadian timing systems have been shown to be regulated by melatonin in this tissue (Wagner et al., 2007), and these seemingly regulate a key output gene, thyroid stimulating hormone β subunit (TSHβ, Fig. 2). Seasonal cycles in the abundance of TSHβ immunoreactivity in the *pars tuberalis* of the Siberian hamster and in its exocytotic activity had been identified a number of years ago (Merks et al., 1993; Wittkowski et al., 1988), but more recent studies in sheep have demonstrated that long days and the consequent short nocturnal melatonin durations result in substantial upregulation of TSHβ in this tissue (Hanon et al., 2008). Cells in the ependymal layer express the TSH-receptor, and local infusion of TSH into the third ventricle has been shown to upregulate DIO2 expression in sheep (Hanon et al., 2008), Siberian hamsters (Klosen et al., 2013), and in Japanese quail (Nakao et al., 2008), so TSHβ appears to be a key paracrine signal released by the *pars tuberalis* that acts locally to effect photoperiodic changes in gene expression in the ependyma. Although this TSHβ pathway has been most comprehensively described in species where seasonal regulation of reproduction has been the focus (sheep, quail), long-day induced up-regulation of TSHβ in the *pars tuberalis* and expression of the TSH-receptor have been identified in many species which display clear annual rhythms in appetite and body weight, for example the Siberian (Wittkowski et al., 1988) and European hamsters (Hanon et al., 2010). It seems highly likely that regulation of DIO2 by TSHβ underlies seasonal cyclicity in both the reproductive and metabolic axes, indeed a recent study in Siberian hamsters maintained in short days has demonstrated that intracerebroventricular infusion of TSHβ increases body weight in addition to promoting testicular recrudescence (Klosen et al., 2013). However, there are also phases of the annual photoperiodic cycle where expression in DIO2 increases despite no evidence for increases in TSHβ expression, such as in hamsters, which reinitiate body weight gain after a prolonged exposure to short days (Bockers et al., 1997; Herwig et al., 2013). This suggests that there are additional signals yet to be identified that are released by the *pars tuberalis* and also regulate gene expression in the ependymal cell layer.

### Experimental tests of the role of hypothalamic thyroid hormone availability

Changes in expression of DIO2 and DIO3 per se are not sufficient evidence that changes in thyroid hormone metabolism regulate hypothalamic function. However, the importance of the thyroid gland and peripheral thyroid hormone concentrations in regulating annual cycles of reproductive activity has been appreciated since the 1930s, as thyroidectomy was shown to prevent seasonal gonadal regression of starlings and ducks, as reviewed by Bechtold and Loudon (2007). A series of studies from the Follett and Karsch laboratories in the 1990s demonstrated a similar dependence on thyroid hormone for seasonal reproductive cycles in sheep. For example, removal of the thyroid gland blocked the long-day induced transition into the seasonal anestrous period (Dahl et al., 1994; Moenter et al., 1991; Parkinson and Follett, 1995), but concurrent treatment with thyroxine allowed the normal timing of reproductive quiescence to occur (Dahl et al., 1995; Webster et al., 1991). These detailed studies provided clear evidence that normal thyroid function provided a permissive signal to allow seasonal neuroendocrine transitions to occur, but the evidence that seasonal cycles of thyroid hormone production provided a specific signal to time transitions was weak. Although low amplitude seasonal variations in circulating thyroid hormone concentrations have been reported in sheep (Lincoln et al., 1980; Webster et al., 1991) with higher levels in winter, these may relate to cold environmental temperatures as much as to short photoperiods. Some studies in Syrian and Siberian hamsters indicate that short-photoperiods or melatonin treatments slightly decrease thyroid hormone levels, but there is inconsistency between studies (Champney, 2001; Masuda and Oishi, 1989). Importantly, at no point of the seasonal cycle do sheep or hamsters become hypothyroid, so it seems very unlikely that cycles in thyroid hormone levels in the peripheral circulation time other seasonal cycles in mammals. However, the evidence reviewed above for photoperiod-regulated changes in DIO2 and DIO3 expression in the ependymal cell layer provides a mechanistic explanation for the earlier observations of dependence on thyroid hormone, in that they would be expected to generate seasonal changes in thyroid hormone availability locally in the hypothalamus. In fact this has been difficult to demonstrate directly, probably because of the technical difficulty of measuring picomolar concentrations of hormone in very localized tissue areas. One study in photoperiodic rats attempted to measure T4 and T3 in pooled whole hypothalamic blocks, but only detected marginally higher T3 levels in LD as compared to SD (Ross et al., 2011).

Studies have now been carried out in Siberian hamsters to determine the functional significance of seasonal regulation of deiodinase gene expression. One experimental approach has been to place microimplants that release thyroid hormone locally within the hypothalamus to determine whether these reverse the changes in physiology and behavior associated with seasonally decreased DIO2/increased DIO3 expression, which would be expected to reduce endogenous concentrations of T3 in the hypothalamus. This approach was based upon that used by Anderson et al. (2003) who demonstrated that such implants resulted in the cessation of the breeding season in female sheep that had previously been thyroidectomized and thus would have otherwise been unable to terminate the reproductive season. In one study, microimplants containing approximately 10 μg of hormone and estimated to release 100 pg of T3 per day were placed bilaterally into hamsters maintained in LD. These T3-implanted hamsters were then transferred to SD. Whereas hamsters with sham implants displayed the expected SD response of reduced food intake, body weight loss, a decrease in intra-abdominal fat depots, and testicular regression, these catabolic processes were completely prevented in hamsters with hypothalamic T3 implants (Barrett et al., 2007). Correspondingly, systemic treatment of Siberian hamsters with daily subcutaneous injections of T3 was also shown to block short-day induced testicular regression (Freeman et al., 2007). These T3 treatments did not, however, interfere with the ability of the hamsters to detect photoperiod, since the pelage of the hamsters exposed to SD molted to a white winter coat (Barrett et al., 2007). Thus, seasonal pelage cycles are not under the control of hypothalamic T3 availability, consistent with earlier studies demonstrating that the winter molt in hamsters results from a SD-induced decrease in prolactin secretion from the pituitary gland (Duncan and Goldman, 1984), presumably driven directly by paracrine signals (“tuberalins”: possibly tachykinin peptides) from the *pars tuberalis* (Dupre et al., 2010).

**Fig. 3:**
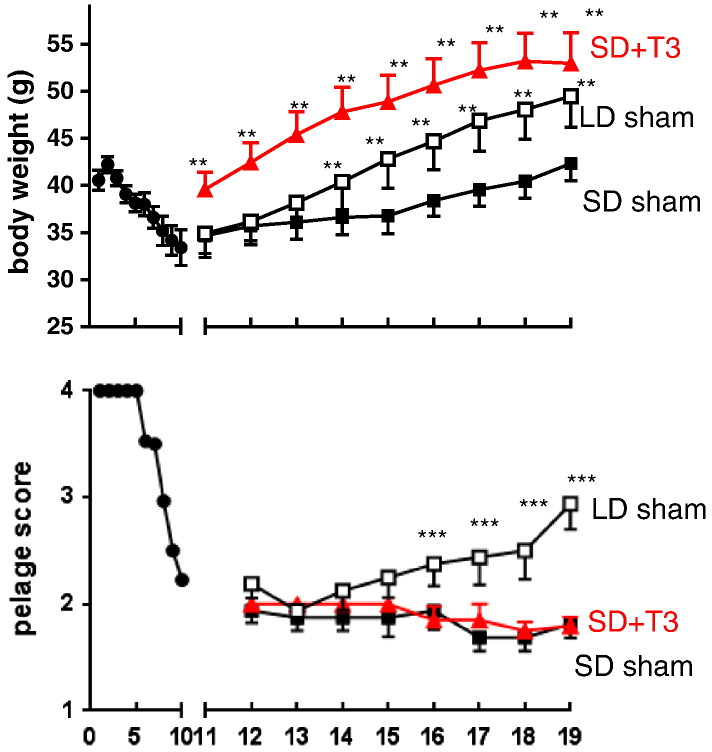
Effects of intrahypothalamic microimplants releasing tri-iodothyronine (T3) on body weight (top) and pelage score (bottom) in male Siberian hamsters in short days (SD). All hamsters were initially maintained on SD for 10 weeks, then received T3 (SD + T3, n = 8) or sham implants (SD sham, n = 8). A third group of hamsters previously exposed to short days for 10 weeks received sham implants but were transferred to long days (LD sham, n = 9). Note that SD initially induced weight loss and a molt to winter pelage (score 1) in all hamsters, and that in those hamsters maintained in SD with sham implants there was then a very gradual increase in body weight reflecting the underlying circannual rhythmicity/photorefractoriness. Implantation of T3 after 10 weeks in SD significantly increased body weight, and the response was more rapid than in sham-implanted hamsters transferred to LD, but the T3 implants did not affect pelage.

Modified from Murphy et al. (2012).

In a complementary experiment, hamsters were exposed to SD for 11 weeks to induce weight loss, and then received intrahypothalamic T3 implants (Fig. 3). These hamsters continued to be kept in SD, but the central T3 treatment promoted a rapid anabolic response, including increased food intake and a gain in body weight (Fig. 3) as compared to controls with sham implants (Murphy et al., 2012). The increase in body weight was not only comparable to that in sham-implanted hamsters transferred to LD as a positive control, but also the onset of response was considerably more rapid (Murphy et al., 2012). Consistent with the earlier study, the T3 treatment had no effect on the pelage (Fig. 3) (Murphy et al., 2012). Systemic T3 treatment has also been shown to promote testicular recrudescence in hamsters previously exposed to short days (Freeman et al., 2007; Henson et al., 2013a), however in neither of these systemic studies did the exogenous T3 promote increased body weight. This dissociation of the actions of thyroid hormone on reproductive and metabolic axes when delivered peripherally may indicate separate sites and/or mechanisms of action. A third study used intrahypothalamic T3 implants in the same experimental paradigm, but hamsters were castrated and also implanted with radiotelemetry devices so that core body temperature could be remotely monitored while the animals remained in their home cages (Murphy et al., 2012). Whereas hamsters with sham implants showed the expected daily torpor bouts in SD that are a characteristic winter adaptation in this species, torpor bouts did not occur in hamsters bearing intrahypothalamic T3 implants, demonstrating that local thyroid hormone availability is also key to this aspect of seasonal energy metabolism (Murphy et al., 2012).

### Mechanisms of action of thyroid hormone and retinoic acid

It is well established that in the periphery, stimulation of thyroid gland activity by the cold, or direct administration of T3, promotes thermogenesis and catabolic activity (Tomasi and Horwitz, 1987). It may seem counterintuitive that increased levels of thyroid hormone centrally promote anabolic responses. However, this is not without precedent, for example, central administration of T3 promotes food intake in rats (Kong et al., 2004). One possible explanation for the experimental findings described above is that hypothalamic implantation of T3 stimulates a negative feedback loop such that suppression of the central control of the hypothalamo-pituitary–thyroid (HPT) axis occurs, and animals are systemically hypothyroid. However, a number of studies describing effects of intracerebroventricular T3 infusions on sympathetic innervation of the liver and brown fat found that such treatments do not alter circulating T3 concentrations (Klieverik et al., 2009; López et al., 2010), so there is little evidence to support the conjecture that the central effects of T3 administration are indirect effects via feedback suppression of the HPT axis. Correspondingly, we have not observed differences in thyrotropin releasing hormone (TRH) mRNA abundance in the hypothalamus of hamsters in the LD and SD states when hypothalamic T3 availability would be predicted to differ (Ebling et al., 2008), so it seems likely that the central actions of thyroid hormone on seasonal cycles are unrelated to control of the peripheral thyroid axis. Given the myriad of roles that thyroid hormone plays in the initial development of the brain, it is tempting to speculate that T3 affects seasonal hypothalamic function via an influence on long-term structural and functional plasticity, rather than by acutely altering the synthesis and secretion of TRH or other neuropeptides.

Actions of T3 have been described in many aspects of the initial development of the brain, from neurogenesis, migration and differentiation of cells to synaptogenesis and myelination (Bernal, 2007). This critical influence of T3 on the maturing brain is harshly illustrated by the severe neurological dysfunction in individuals with genetic mutations of the monocarboxylate 8 transporter that is essential for cellular transport of T3 (Dumitrescu et al., 2004; Friesema et al., 2004). It is becoming increasingly apparent that many of these processes persist at a low level in the adult hypothalamus, raising the question of whether seasonal cycles of growth, body weight and reproduction are a recapitulation of these early developmental mechanisms (Hazlerigg and Lincoln, 2011).

Several lines of evidence support this conjecture. First, seasonal plasticity in synaptic appositions on GnRH terminals has been identified in sheep, probably reflecting retraction of ensheathing tanycyte end feet (Xiong et al., 1997). Correspondingly, analysis of vimentin immunoreactivity in Siberian hamsters has revealed photoperiodic effects on the abundance and morphology of tanycyte processes (Bolborea et al., 2011; Kameda et al., 2003). Second, there is now abundant evidence of cell division in the mediobasal hypothalamus of mammals (Kokoeva et al., 2007; Xu et al., 2005). In non-seasonal rodents the rate of cell division has been shown to be modifiable by diet, though there is no consensus as to what phenotype such cells differentiate into, nor what the underlying mechanisms promoting division are, with reports that high fat diets can both increase (Lee et al., 2012) and decrease (McNay et al., 2012) neurogenesis. In hamsters (Huang et al., 1998) and sheep (Hazlerigg et al., 2013) seasonal increases in cell division inferred from differential uptake of the thymidine analog BrdU have been observed in the hippocampus, though this has not been observed in gray squirrels (Lavenex et al., 2000), and there is one report that rates of division in the hypothalamus are greater in winter as compared to summer (Migaud et al., 2011).

Many questions remain as to the functional significance of these observations. There is some disagreement as to whether the dividing cells in the hypothalamus really differentiate into neurons. Whereas one study in sheep reported that approximately 17% of BrdU positive cells in the hypothalamus co-expressed the neuronal marker Neu-N (Migaud et al., 2011), another study failed to find evidence that dividing cells adopted a neuronal phenotype in this region (Hazlerigg et al., 2013). Interesting, from other model systems there is some evidence that blockade of cell division in the mediobasal hypothalamus using anti-mitotic compounds or irradiation ameliorated leptin- (Kokoeva et al., 2005) and high fat diet- (Lee et al., 2012) induced changes in metabolism, suggesting a causal role for cell division in hypothalamic function. It remains to be determined whether thyroid hormone is the critical regulator of seasonal plasticity in the adult hypothalamus. Because thyroid hormone is the critical regulator for neurogenesis in the subventricular zone of mice (Lemkine et al., 2005), this remains a very attractive hypothesis to test using mammals that vary seasonally in appetite and body weight.

### Thyroid hormone and reproductive cycles

The original studies utilizing thyroidectomy and more recent experiments using central and peripheral administration of thyroid hormone all demonstrate the strong link between thyroid hormone availability and reproductive function. As control of gonadotropin secretion in mammals is entirely dependent on the secretion of GnRH (actually mGnRH1) from a few hundred neurons in the basal forebrain (Wray, 2010), study of this axis may provide a more tractable approach for understanding the cellular actions of thyroid hormone. As noted above, there is some evidence from studies in sheep for seasonal plasticity of glial ensheathment of GnRH terminals (Xiong et al., 1997), and also for seasonal changes in density of synaptic appositions onto GnRH soma and dendrites (Adams et al., 2006). However, a widely accepted view is that GnRH neurons are rather passive components of the reproductive axis, and that physiological changes in GnRH secretion, for example at puberty, across the menstrual/estrous cycle, and seasonally, reflect altered inputs to GnRH neurons (Ebling, 2005).

High in the hierarchy of such inputs to GnRH neurons is the kisspeptin system, since the cognate GPR54 receptor is one of the few receptors to be abundantly expressed by GnRH neurons (Messager et al., 2005), and because congenital absence of kisspeptin or GPR54 in man or targeted ablation of these in mice results in complete infertility (d'Anglemont de Tassigny et al., 2007; Seminara et al., 2003). This hypothalamic system is regulated by photoperiod in seasonal species: short days reduce the population of kisspeptin immunoreactive neurons in the anteroventral periventricular nucleus (AVPV) of male and female Siberian hamsters (Greives et al., 2007; Mason et al., 2007). Correspondingly Kiss1 mRNA abundance is decreased in the arcuate nucleus of male Syrian hamsters exposed to short days, a pineal dependent process (Revel et al., 2006a). The short day-induced decrease in kisspeptin immunoreactive neurons in AVPV is partially a reflection of decrease circulating testosterone levels, but importantly decreases are also seen in castrate hamsters, demonstrating a direct photoperiodic effect (Greives et al., 2008). Likewise, in female sheep there is a decrease in kisspeptin-immunoreactive neurons in the hypothalamus in the anestrous season in both intact and ovariectomized ewes, suggesting fundamental photoperiodic regulation of this system [reviewed by Clarke, 2014]. One antiserum used to detect kisspeptin neurons cross reacts with RFamide-related peptides 1 and 3 (RFRP1, RFRP3), as the peptides share a common amidated arginine–phenylalanine sequence at their C-termini. RFRP is also photoperiodically regulated in the dorsomedial nucleus of the hypothalamus, and in most experimental paradigms is an inhibitor of GnRH release, hence its alternative name GnIH (GnRH-inhibitory hormone) (Kriegsfeld et al., 2006). However, the direction of change of RFRP in short photoperiods has not been found to be consisted between laboratories and species, with some studies demonstrating an upregulation in the dorsomedial nucleus of Siberian hamsters (Paul et al., 2009), but others finding a downregulation in this species and also in Syrian hamsters (Revel et al., 2008). To add complexity, whereas RFRP reduces gonadotropin secretion in hamsters in the long-day state, it can induce secretion in the short day state (Ubuka et al., 2012). Seasonal regulation of these RFamide peptides have also been found in sheep, but with some regional differences compared to rodents. For example, short days increase the number of kisspeptin-immunoreactive cells in the arcuate nucleus of sheep, an effect independent of circulating estradiol concentrations (Smith et al., 2008). Correspondingly, RFRP immunoreactivity was decreased in dorsomedial nucleus of the hypothalamus, consistent with the reproductive status of the sheep (Smith et al., 2008). Intriguingly, a separate study revealed that the ependymal cell layer also expresses RFRP, and this is down regulated in short days when the sheep are reproductively active (Dardente et al., 2008). The important point is that RFamide systems are photoperiodically regulated in seasonal mammals, and are powerful regulators of GnRH/gonadotropin release (Simonneaux et al., 2013; Tsutsui et al., 2013), so are likely to be targets of thyroid hormone signaling. Two recent studies provide evidence that this is indeed the case, as treatment of Siberian hamsters in short days with either intracerebroventricular TSHβ (Klosen et al., 2013) or systemic thyroid hormone (Henson et al., 2013b) induced testicular recrudescence, which was associated with induction of long-day patterns of kisspeptin and RFRP immunoreactivity.

## Conclusion

Seasonal cycles of adiposity reflect programmed changes in appetitive and consummatory behaviors. The investigation of the central mechanisms underlying these behaviors has undoubtedly led to some novel insights into hypothalamic control of food intake. Hypothalamic tanycytes have been revealed as key determinants of long-term changes in ingestive behavior and energy metabolism through their role in transport and regulation of thyroid hormone availability in the hypothalamus, and perhaps of other developmental signals such as retinoic acid. The mechanisms by which thyroid hormone regulates seasonal cyclicity remain to be elucidated, but it seems likely that they involve the recapitulation of mechanisms important in initial development, and perhaps also actions on RFamide peptidergic systems in the hypothalamus. We should therefore view the hypothalamus as a plastic region of the brain, capable of being reprogrammed, and should recognize that seasonal species provide valuable model systems to elucidate the mechanisms underlying such plasticity.

## Figures and Tables

**Table 1:** Effects of short day photoperiod on the expression of deiodinases in the ependymal layer of the third ventricle.

Species	DIO2	DIO3	References
Siberian hamster *Phodopus sungorus*			Barrett et al. (2007)Watanabe et al. (2004) Kampf-Lassin and Prendergast (2013), Prendergast et al. (2013)
Syrian hamster *Mesocricetus auratus*		n/a	Revel et al. (2006b)
European hamster *Cricetus cricetus*			Hanon et al. (2010)
Common vole *Microtus arvalis*			Król et al. (2012)
Fischer F344 rat^a^ *Rattus norvegicus*			Ross et al. (2011) Yasuo et al. (2007)
Sheep *Ovis aries*			Hanon et al. (2008) Sáenz de Miera et al. (2013)
CBA/N mouse *Mus musculus*			Ono et al. (2008)

–: upregulated, –: downregulated, n/a: data not available.

a Juvenile males.
